# Tri-model classifiers for EEG based mental task classification: hybrid optimization assisted framework

**DOI:** 10.1186/s12859-023-05544-1

**Published:** 2023-10-30

**Authors:** Awwab Mohammad, Farheen Siddiqui, M. Afshar Alam, Sheikh Mohammad Idrees

**Affiliations:** 1 Department of Computer Science and Engineering, Jamia Hamdard, New Delhi, New Delhi, 110062 India; 2 Department of Computer Science (IDI), Norwegian University of Science and Technology, 2815 Gjøvik, Norway

**Keywords:** Emotions, Proposed DBN, Improved entropy, Optimal weight, SSU-BES algorithm

## Abstract

The commercial adoption of BCI technologies for both clinical and non-clinical applications is drawing scientists to the creation of wearable devices for daily living. Emotions are essential to human existence and have a significant impact on thinking. Emotion is frequently linked to rational decision-making, perception, interpersonal interaction, and even basic human intellect. The requirement for trustworthy and implementable methods for the detection of individual emotional responses is needed with rising attention of the scientific community towards the establishment of some significant emotional connections among people and computers. This work introduces EEG recognition model, where the input signal is pre-processed using band pass filter. Then, the features like discrete wavelet transform (DWT), band power, spectral flatness, and improved Entropy are extracted. Further, for recognition, tri-classifiers like long short term memory (LSTM), improved deep belief network (DBN) and recurrent neural network (RNN) are used. Also to enhance tri-model classifier performance, the weights of LSTM, improved DBN, and RNN are tuned by model named as shark smell updated BES optimization (SSU-BES). Finally, the perfection of SSU-BES is demonstrated over diverse metrics.

## Introduction

Many neuron-based systems have been developed to discuss solutions for prediction of diseases, their communication, and control via using a BCI system. Supervised learning, unsupervised learning, and semi-supervised learning are the most often utilized methodologies. A machine learning expert who is familiar with preconditioned feature extraction techniques is required.

Furthermore, the key challenge is selecting useful features for solving an issue from a challenging assignment. Deep learning techniques for a specific problem can bypass the burden of extracting useful features from raw input data for feature selection. A multi-level network is required to learn numerous specific features.

Image identification, audio recognition, language translation, natural language understanding, signal processing, face recognition, and other applications benefit from encouraging findings obtained through deep learning. We need to measure and analyze brain signals in a standard BCI workflow to get any usable outputs for computers to read. EEG is used to assess the electrical activity of various brain areas. Because testing and working on EEG signals is extremely difficult and exhausting, an automated computer-aided diagnosis approach must be created. When we categorize patterns using sampled waveforms, we get terrible results. To improve classification performance, we must extract only the data's distinguishing characteristics. Human–computer interaction (HCI) [1] technologies are already present in many aspects of our daily life. The rapid advancement of HCI technology and its applications in several industries have sparked a significant deal of interest in creating HCI that is more intelligent. Without a doubt, the development of humanized human-machine interfaces in the field of HCI depends on human emotions.

Emotions are most unique features of people, and they have an impact on how they behave and act. An essential component of human life is comprehending and understanding emotions [2, 3]. Human emotions, ideas, and conduct are reflected in emotion. In the fields of remote learning, healthcare, and HCI, emotion detection is a popular study area [4, 5]. For instance, in medical care, identifying the emotions of patients, particularly those with disorders of expression, allows one to take various nursing measures in accordance with the patient’s emotions and enhance nursing quality [6–8]. The EEG, a traditional physiological signal, is quick and impervious to human control. As a result, it has been frequently used in treating with emotion identification [9]. The majority of the earlier EEG emotion identification methods concentrate on two fundamental problems:

1. How to identify distinguishable emotional EEG signals
2. How to create a more powerful model for emotion identification [10–12]. The majority of techniques for retrieving exclusionary EEG emotional features fall into one of three categories: frequency, time or frequency-time domain (e.g., DWT) [13, 14].

However, as EEG signal is non- stationary, non-linear, and includes a substantial amount of noise, the field of emotion identification based on signal properties is exceedingly difficult. Additionally, the EEG signal's features are primarily retrieved from its frequency, time or frequency–time domain, as well as its spatial domain [15–18]. The asymmetry among electrode pairs has received a lot of attention from scientists who are building schemes for identifying emotions due to the spatial properties. In other words, the techniques typically relate to the variations in signals detected by the matching electrodes on right and left hemisphere of the brain, accordingly.

The contributions are as follows:

- Determines improved entropy along with spectral flatness, band power and DWT.
- Introduces tri-classifiers (LSTM, DBN and RNN) classifiers with optimization strategy for recognition purpose.
- Proposes SSU-BES algorithm for training the tri-classifiers via tuning the optimal weights, which ensures the efficiency in emotion recognition.

Here, Sect. 2 analyses the related work. Section 3 offers explanation on novel EEG based emotion recognition approach. Section 4 and 5 depicts features and tri-classifiers. Section 6 depicts about SSU-BES based optimal weight election and results are given in Section 7 and 8.

## Literature survey

### Related work

Yishu [19] suggested learning multi-channel characteristics from EEG signal in 2021 to recognize human emotions, with the EEG data being triggered by sound signals. To identify various human emotions, they specifically employed textual feature fusion and multi-channel EEG in the time domain, wherein, 6 characteristics in the time domain were merged to a feature set for emotion categorization. Based on text, textual features were extracted. Additionally, they extracted EEG and text based features in the temporal and frequency domains. Lastly, they trained SVM to recognize human emotions. Their suggested technique increases detection accuracy rate according to experiments on the DEAP dataset.

Abdul Hamit [20] introduced an automated approach for recognizing emotions using EEG data. The suggested procedure was simple and involves four main steps. During the pre-processing stage, a DWT-based noise reduction approach was used. As a feature extractor, a TQWT was used. For dimension reduction, there are six alternative statistical techniques. The RFE classifier was used in the classification step in conjunction with a variety of classification algorithms, including KNN, SVM, ANN, RF, and 4 distinct DT algorithms. The findings unequivocally demonstrated the viability of the suggested TQWT and RFE driven emotion identification framework as a method for identifying emotions from EEG data.

A BiDCNN for EEG emotional identification was proposed by Dongmin et al. in 2021 [21] and could successfully learn the various response patterns between the right and left hemispheres. Three separate EEG feature matrix were built specifically to record and magnify various electrical brain reactions to emotional stimuli. The spatial and temporal data were then retrieved using 3 CNN layers. The subject independent experimental findings further demonstrated that BiDCNN achieved superior outcomes on the valence and arousal detection tasks, with accuracy rates of 68.14 % and 63.94 %, respectively. Different participants were utilized to train and test the model.

The development of extracted features depending on HOLO-FM presentation of EEG signal characteristics was the foundation of work proposed by Ante [22] for emotion identification. On feature maps, DL was employed as a feature extractor approach. Extracted features were then combined for recognition system to recognize various types of emotions. By comparing their methods to existing studies in which the authors used EEG data to categorize human emotions in a two-dimensional space, they were able to show that their method was more successful. The suggested approach could increase the emotion identification rate on datasets of various sizes, according to experimental findings.

Wenjing [23] updated the correlation mode and extracted characteristics for emotion identification study in 2021 using CFC. Their findings demonstrated that the CFC-based EEG network outperformed existing EEG synchronization networks in the categorization of emotions. Additionally, combining local and global characteristics as well as dynamic network characteristics can significantly increase emotion identification accuracy. This investigation made a ground-breaking exploration for future studies on selecting features of emotion detection and connected neuronal pathways of functional interconnections and introduces a different idea of data connection for the further research of emotion detection and other enhanced cognitive actions.

A unique scaling layer that may mine useful data-driven spectrogram-like characteristics from unprocessed EEG signals was proposed by Hu *et al.* in 2021 [24]. In addition, it used convolution kernels that have been scaled up from a single data driven prototype to reveal a frequency-like size, which helped it to overcome the drawbacks of previous existing approaches that required retrieving the characteristics and their estimates manually. The suggested neural network architecture called Scaling Net, was dependent on Scaling Layer that produced cutting-edge results on the well-known datasets.

In 2020, Yu Liu [25] suggested an efficient scheme for emotion identification. It was a complete framework that could concurrently identify emotional states and extract features from unprocessed EEG data. When creating the principal capsules, it integrated multi-level feature mappings learnt by several layers in comparison to the original Caps Net, enhancing the ability of feature representation. In order to decrease the number of constraints and speed up computation, it also employed a bottleneck layer. The findings demonstrated that their method was more accurate than cutting-edge techniques.

A unique fractal pattern feature based method was described by Turker *et al.* [26] combined with an automated EEG-based emotion identification algorithm. A multi-layer feature generator was proposed utilizing FFP and TQWT signal decomposing approach. An enhanced iterative selector was used throughout the feature selection step. The effectiveness of the provided FFP and TQWT feature creation has been thought to be shown by the shallow classifiers. This model has been evaluated on 14-channel emotional EEG data using SVM, LDA, and KNN. With SVM, the suggested framework obtained 99.82 percent.

Empirical selection of decomposition parameters can result in information loss owing to mode mixing, production of noisy modes, inadequate signal synthesis, and so on. Optimized variational mode decomposition was proposed in the study by Smith K Khare *et al.* [27] for emotion recognition utilizing single-channel EEG recordings. For dominating channel selection, the Eigenvector centrality technique (EVCM) is used. For non-stationary EEG signal decomposition, an optimal number of modes (K opt) and penalty factor (opt) are chosen adaptively. The modes of EEG signals are used to extract time-domain information. Post-hoc analysis is performed to pick relevant characteristics, which are then used as input to various classifiers for emotion classification. An extreme learning machine classifier achieves an overall accuracy of 97.24% for a four emotion classification. Five performance parameters are used to assess the suggested method's performance. The F-1 score, false-positive rate, Mathew's correlation coefficient, and Cohen's Kappa are calculated to be 0.9454, 0.94%, 92.92%, and 0.9633, respectively. Using the same dataset as the classic variational mode decomposition and the existing state-of-the-art, the using the same dataset, the proposed method shows improved performance of about 4% and 2%.

Emotions are the most potent information source for studying a person's cognition, behavior, and medical issues. Accurate emotion recognition aids in the development of emotional computing, brain-computer interfaces, medical diagnosis systems, and so on. EEG signals are one such source for capturing and studying human emotions. The authors Smith K Khare *et al.* [28] proposed a unique time-order representation for identifying human emotions that is based on the S-transform and a convolutional neural network (CNN). The S-transform is used to translate EEG signals into time-order representation (TOR). This TOR is fed into CNN, which automatically extracts and classifies the deep features. The classification of emotional states of happiness, fear, sadness, and relaxation is 94.58% accurate. The method's superiority is determined by evaluating four performance parameters and comparing it to existing state-of-the-art on the same dataset.

Convolutional networks and recurrent networks are the traditional approaches for learning complicated spatial connections over several electrodes and brain regions. Yet, due to the processes of local feature learning, these models have trouble capturing long-range relationships. Z. Wang *et al.* [29] proposed a transformer-based approach to hierarchically learn discriminative spatial information from electrode level to brain-region level to improve EEG spatial dependency capture and emotion detection accuracy. Transformer encoders are used in electrode-level spatial learning to integrate information across multiple brain areas. A transformer encoder is used in brain-region-level spatial learning to record spatial interdependence between brain regions. Finally, subject-independent tests are performed on the DEAP and MAHNOB-HCI databases to confirm the usefulness of the proposed model. The experimental findings show that the suggested model performs very well in emotion recognition with arousal and valence level. Furthermore, the visualization of self-attention suggests that the suggested model may prioritize discriminative spatial information from the pre-frontal, frontal, temporal, and parietal lobes.

Apart from the usage of dry electrodes and wireless technologies, minimizing the number of channels is critical for improving device ergonomics. The study of A. Apicella *et al.* [30] provides a review of research that used fewer than 16 channels for EEG-based emotion identification. The main findings of this review are as follows:

1. The guidelines for choosing the most promising scalp areas for EEG acquisitions;
2. The importance of prior neurophysiological knowledge;
3. The convergences between different studies in terms of preferable scalp areas for signal acquisition.

For channel selection, three ways emerge: data-driven, previous knowledge-based, and based on commercially available wearable solutions. The most common is data-driven, although the neurophysiology of emotions is rarely considered. Additionally, commercial EEG equipment seldom has electrodes that have been specifically selected to evaluate emotions. Several electrodes show significant convergences: Fp1, Fp2, F3, and F4 are the most informative channels for the valence dimension, according to both data-driven and neurophysiological prior knowledge techniques. P3 and P4 were shown to be relevant for the arousal component.

In the past few decades, EEG-based emotion recognition has emerged as one of the most important issues in the domains of healthcare, education, information sharing, gaming, and many others. In order to predict six fundamental emotions, Md. Mustafizur Rahman *et al.* [31] suggested three non-linear characteristics and eight ensemble learning methods (hope, interest, excitement, shame, fear, and sad). They used a randomized grid search method to fine-tune each algorithm's hyper-parameter in order to boost the recognition rate of each classifier. To evaluate the effectiveness of each approach, they ran all the tests on the DEAP and AMIGOS datasets and assessed the calculation time and accuracy. Also, they examined statistical significance to evaluate the effectiveness of the method. According to the experimental findings, they used the Higuchi fractal dimension on the DEAP and AMIGOS datasets, respectively, to reach the greatest average accuracy, 89.38% and 94.62%. Comparing their suggested methodology to current methods that used the same dataset, the average recognition rate rose by 8.22% and 1.77%, respectively.

### Review

Regarding adaptability, different ML algorithms are obtainable for use in a range of applications and, thanks to technical advances in computing and effective application of algorithms, they can complete complex calculations with little time wasted [8]. However, there is no proof that any one algorithm is superior to others, which makes choosing an algorithm for emotion categorization assignments is challenging. Additionally, in terms of adaptability, a trained model for ML algorithms which may be utilized for bench marking or commercial deployment for upcoming emotion classifications is required. According to a survey of recent works, the authors have attempted improvements to CNN [21] in this area, but more research on domain agnostic emotion subspace is required. Similar to how ensemble models have advanced; the authors have developed an emotion categorization approach that requires more certainty in handling large datasets [20]. SVM [19] is now used to verify the effectiveness of categorizing emotions using deep textual and EEG feature training. This has shown acceptable results for categorizing emotions. The model must be created to operate with actual datasets, though. The feature maps in [22] were created by the authors using TOPO-FM representations, and DL model was deployed to take out features. This model has demonstrated its superiority in categorizing emotions; nevertheless, for improved outcomes in practice, the categorization should be tested using additional feature sets and cross validation. In order to make the categorization more accurate in practice, more algorithms and feature extractors are required because the advancement is not sufficiently exact in all of its features.

## Explanation on novel EEG based emotion recognition approach

This work introduces a new EEG oriented emotion recognition model with subsequent stages.

- At first, band pass filtering is used to pre-process the input signal.
- Then, DWT, band power, spectral flatness, and improved entropy based features are extracted.
- Then, tri-model classifiers is determined that includes LSTM, Improved DBN and RNN for recognizing the emotions from the signal. Here, the training process takes place under optimization scheme via optimal weight tuning of the classifiers.
- Introduces a new SSU-BES algorithm for resolving the searching ability of BES with SSO.

**Fig. 1:**
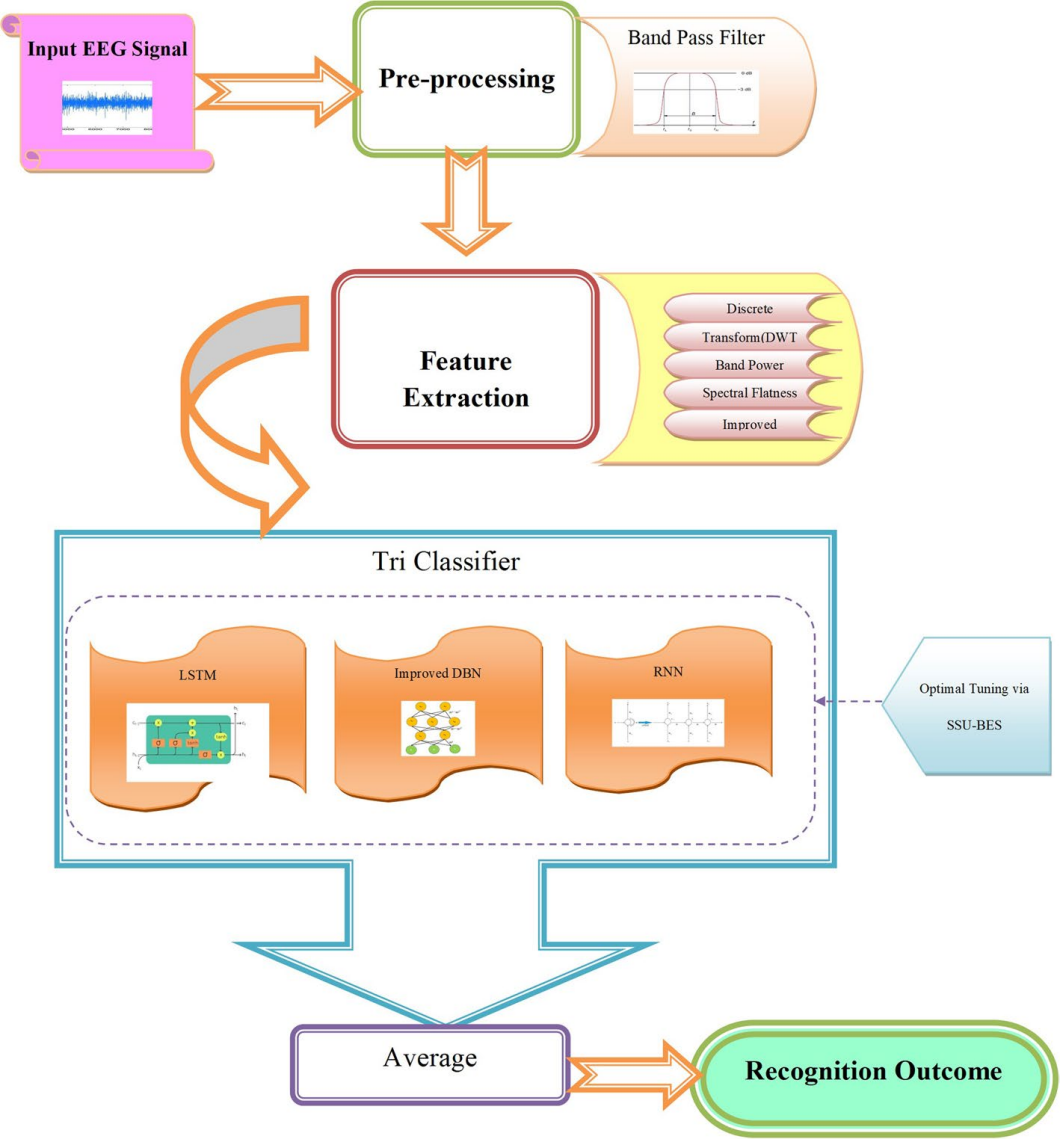
Pictorial model of SSU-BES method

The illustrative depiction of SSU-BES model is exposed in Fig. 1.

### Pre-processing

Let the input signal be S, which is subjected for pre-processing phase. This is the initial phase before classifying it. In this work, BPF based pre-processing takes place. In general, BPF [32] is a circuit or device that eliminates (attenuates) frequencies outside of a specific range while passing frequencies inside of that range A band-pass filter inhibits elements with frequency range below or above its pass band while allowing components within a predefined range of frequencies to pass through. The pre-processed signal is denoted as Spre.

## DWT, band power, spectral flatness, and improved entropy features

Subsequent to the pre-processing, the feature extraction process takes place, where the relevant features are extracted from the pre-processed signal. In this work, we are determining few features during the extraction, and that are as follows:

- DWT
- Band power
- Spectral flatness
- Improved entropy

### DWT features

The WT theory requires an evaluation of signals at various frequencies and timings. A wavelet is actually a harmonics with an average mean of zero and a practically fixed period. The CWT divides a time domain function into wavelets. However, CWT implementation is constrained by data redundancy and the considerable amount of computation needed to compute all practical transformations and scales. The DWT improves the WT [33], which boosted the deconstruction method's adaptability. An orthogonal DWT can be used to evaluate a given collection of data for several scales. Eq. (1) provides the mathematical formula for the DWT.

1 $$DWT(g,v) = \int\limits_{ - \infty }^{ + \infty } {x(t)*\tau_{u,v} \left( g \right)dt}$$

The arithmetical model for mother wavelet is shown in Eq. (2) and $v$&$g$ signifies scale and shift coefficients.

2 $$\tau_{u,v} \left( g \right) = \frac{1}{\sqrt g }\tau \left( {\frac{g - v}{g}} \right)$$

### Band power

Decomposing the signal into functionally separate frequency bands, such as delta (0.5–4 Hz), theta (4–8 Hz), alpha (8–12 Hz), beta (12–30 Hz), and gamma (30–100 Hz) [34], is one of the most used techniques for analysing EEG data. The lower frequency bands (theta and delta), will have bigger exponential values than higher frequency bands (beta and alpha), since the EEG wave bands reflect a power spectrum [35]. They are helpful in their present output form as a measure of each band's strength in relation to the other bands as well as whether that band is growing or shrinking over time [36].

### Spectral flatness

The term Wiener entropy, commonly referred to as spectral flatness or tonality coefficient [37], is a measurement used in digital signal processing to describe an audio spectrum. A sound's spectral flatness, which is commonly expressed in decibels, can be used to gauge how closely it approaches a pure tone as opposed to seeming noise-like. As an alternative to measuring the spectral flatness throughout the whole band, it may also be done within a specific sub band.

The spectral flatness is calculated as revealed in Eq. (3), in which,$y\left( m \right)$ signifies magnitude of bin count *m*.

3 $$SF = \frac{{\sqrt {\prod\nolimits_{m = 0}^{M - 1} {y\left( m \right)} } }}{{\frac{{\sum\nolimits_{m = 0}^{M - 1} {y\left( m \right)} }}{M}}}$$

### Improved entropy

How much information is conveyed by a signal, is explained by entropy. Equation (4) is used to compute entropy. Nevertheless, to resolve the reliability issues, numerous changes are implemented to the current process. Equation (5) shows improved entropy, in which, $b$ → random term and → probability distribution and → EEG signal, $pos$ and $neg$→ positive and negative reviews, $wei_{i}$ → weight that is estimated using Bernoulli map.

4 $$fe_{en} = - \sum\limits_{i = 1}^{b} {P\left( {y_{i} } \right)\log P\left( {y_{i} } \right)}$$

5 $$fe_{Ien} = \frac{{\sum\limits_{i = 1}^{b} {P\left( {pos\left| {y_{i} } \right.} \right)\frac{neg}{{pos}} * \log_{2} In\left( {P\left( {neg\left| {y_{i} } \right.} \right)\frac{pos}{{neg}}} \right)} }}{{wei_{i} }}$$

The extracted feature set $fe$ that includes DWT, SF, fe _Ien_ are then subjected to optimization assisted tri-classifiers (LSTM, DBN and RNN).

## Optimization assisted tri classifiers: LSTM, improved DBN and RNN

This work deploys tri-classifiers (LSTM, Improved DBN and RNN) for recognizing emotions in EEG signal. Here, the tri-classifiers due process in this way: The features extracted are parallel subjected to all the three classifiers. From which, the results are obtained. We can denote that as LSTM^out^, Improved DBN^out^, RNN^out^ respectively. Once after the determination of the individual outcome, all the three are averaged to define the final results. Moreover, during the training process of the classification, the weights of all the three classifiers are tuned optimally by novel SSU-BES algorithm. This is because; it is observed that the optimal tuning ensures high efficiency during the classification, since the training is the crucial part to define the appropriate recognition with respect to features. Hence, during testing, the model could accurately recognize the emotions.

### LSTM

It [36] included: a forget gate, input gate, and output gate. Assume that variables $Y$ and *A* are concealed and cell states. $\left( {D_{t} ,\,A_{t - 1} ,Y_{t - 1} } \right)$ and $\left( {Y_{t} ,\,\,A_{t} } \right)$ be input and output layers. LSTM used $G_{t}$ to arrange the data as revealed by Eq. (6), in which, $\sigma$→ activation function,$\left( {P_{YG} ,G_{YG} } \right)$ and $\left( {P_{IG} ,F_{IG} } \right)$ implied weight and bias constraints to map hidden and input layers to forget gate At time $t,$ the forget gate →$G_{t}$, input gate →$I_{t}$ and output gate →$L_{t}$.

6 $$G_{t} = \sigma \left( {P_{IG} X_{t} + P_{YG} Y_{t - 1} + F_{IG} + F_{YG} } \right)$$

*I*_*t*_ is used in LSTM as in Eqs. (7)–(9), here, $\left( {P_{YV} ,L_{YV} } \right)$ and $\left( {P_{IV} ,V_{IV} } \right)$→ weight and bias factors to map hidden and input layers to cell gate. $\left( {P_{YI} ,F_{YI} } \right)$ and $\left( {P_{II} ,F_{II} } \right)$ imply weight and bias constraint to map hidden and input layers to $I_{t}$. It get output hidden layer from $L_{t}$ as in Eqs. (10) and (11), in which, $\left( {P_{YL} ,F_{YL} } \right)$ and $\left( {P_{IL} ,F_{IL} } \right)$→ weight and bias to map $L_{t}$.

7 $$V_{t} = \tanh \left( {F_{IV} + P_{IV} X_{t} + F_{YV} + P_{YV} Y_{t - 1} } \right)$$

8 $$I_{t} = \sigma \left( {P_{II} X_{t} + P_{YI} Y_{t - 1} + F_{II} + F_{YI} } \right)$$

9 $$A_{t} = G_{t} A_{t - 1} + I_{t} V_{t}$$

10 $$F_{t} = \sigma \left( {L_{IF} + J_{IF} X_{t} + L_{YF} + J_{YF} Y_{t - 1} } \right)$$

11 $$Y_{t} = L_{t} \tanh \left( {A_{t} } \right)$$

### RNN

The variable length input sequence may be handled by RNN [38, 39]. The RNN includes the following 4 weights or biases: Forget gate layer, Input gate layer, Output gate layer and State gate layer. The input and forget gates are in charge of controlling both the current input state and preceding concealed state. The features $\left\{ {fe_{1} ,fe_{2} ....,fe_{M} } \right\}$ are given as input to RNN and the hidden state sequence is implied by $\left\{ {wg_{1} ,wg_{2} ,....,wg_{M} } \right\}$ and the output vector is implied by $\{ I_{1} ,I_{2} , \ldots I_{m} \}$. The hidden state and output is computed as in Eqs. (12) and (13), in which,$rf$ implies recurrent function,$F_{i}$ implies input vectors,$wg_{i}$ implies hidden unit,$P,Q$,$R$ implies weight matrices; and activation function is implied by $\tanh$. The total weights are determined by the new SSU-BES algorithm.

12 $$wg = rf(F_{i} ,wg_{i - 1} ) = \tanh (P.F_{i} + Q.wg_{i - 1} )$$

13 $$I_{i} = soft\max (R,wg_{i} )$$

### Improved DBN

A generative graphical model called DBN [40] or DNN groupings, which consists of multiple layers of hidden units (latent variables). Associative memories make up the two top layers of DBN that are symmetrically linked. The arrows pointing to the layer closest to the data direct relationships to all below layers.

The lowest layer, or the observable units, that's where the input data is collected. Actual input information is an integer. There have been no intra layer interconnections like the RBM. Software that finds similarities in data is known as a hidden unit. The weight matrix $\left( {We} \right)$ that links the two levels is symmetric. Every device in every layer is linked to a higher layer below it.

The pre-training of DBN is done using a contrastive divergence algorithm. In this work, SSU-BES based training is also determined that tunes the weighting parameter. There have the weight connection among each layer. These weights define which variables in 1 layer are used to compute the components in the layer above. In DBN, we examine the top 2 hidden layers of Gibbs using a variety of metrics. Essentially, the RBM represented by 2 top timed layers is sampled in this step. The values of the latent variable may be used to create a basic bottom-up transfer. Here, the recognition outcomes are determined based on the actual and predicted value via evaluating the loss function. As per the improved version, a new evaluation is done for loss calculation that calculates MAEL, which is given in Eq. (14), where $\hat{x}$ and $x$ denote the projected value and actual value, respectively.

14 $$L\left( {x,\hat{x}} \right) = \frac{1}{N}\sum\limits_{i = 0}^{N} {\left[ {x - \hat{x}_{i} } \right)}$$

The results from tri-classifiers are averaged and absolute outcome is accomplished.

## SSU-BES based optimal weight tuning process

**Fig. 2:**
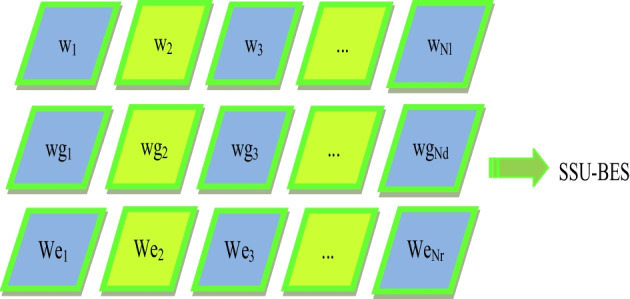
Solution encoding

As mentioned before, the training of weights in classifiers are done by SSU-BES algorithm. The solution given to the algorithm is shown in Fig. 2. During this, the objective fixed is defined as $Obj$ in Eq. (15).

15 $$Obj = \min \left( \frac{1}{accuracy} \right)$$

### Proposed SSU-BES algorithm

A renowned optimization scheme with better convergence is BES [10]. Even so, the performance of the searching ability and swooping stage of BES is poor which leads to pre-mature convergence and falls in local optimum. Thus, a hybrid optimization SSU-BES is developed by merging the SSO in BES. As, the hybridization of algorithms become crucial and efficient, they are applicable to solve complicated search issues [41–44]. The following is a description of mathematical definition of proposed algorithm:

Choosing space stage: This step determines the ideal area based on the amount of food. Eq. (16) accurately models this behaviour.

16 $$Z_{new} = Z_{best} + \beta \times ra\left( {Z_{mean} - Z_{i} } \right)$$

In Eq. (16), $Z_{best}$ refers to elected searching space depending upon best position of eagle, $Z_{mean}$ refers to mean distance amid every positions of bald eagle (population mean), $Z_{i}$ refers to present position of eagle, $ra$ refers to random constraint produced among [0—1], $\beta$ is controlling parameter.

Improved Searching Space stage: The eagle is currently searching for prey by flying in selected spiral space in a variety of orientations. Additionally, the ideal swooping and hunting positions are shown. Traditionally, Eq. (17) provides a detailed definition of this behaviour, nevertheless, as per SSU-BES; this behaviour is computed based upon the combination of SSO update as in Eq. (18), in which $\Re 1$ refers to random parameter,$\eta_{k}$ refers to a value among 0 and 1. In addition, conventionally $\Re 1$ is randomly computed, as per the proposed SSU-BES, $\Re 1$ is computed depending upon circle map.

17 $$Z_{new} = Z_{i} + Y\left( i \right) \times \left( {Z_{i} - Z_{i + 1} } \right) + p\left( i \right) \times \left( {Z_{i} - Z_{mean} } \right)$$

18 $$Z_{new} = \eta_{k} .\Re 1\left( {\frac{{Z_{i} + Y\left( i \right) \times \left( {Z_{i} - Z_{i + 1} } \right)}}{{\left. {\nabla of} \right|_{{\chi_{i,j}^{k} }} }}} \right) + p\left( i \right) \times \left( {Z_{i} - Z_{mean} } \right)$$

19 $$p\left( i \right) = \frac{pr\left( i \right)}{{\max \left( {\left| {pr} \right|} \right.}},\,\,Y\left( i \right) = \frac{Yr\left( i \right)}{{\max \left( {\left| {Yr} \right|} \right.}}$$

20 $$pr\left( i \right) = \cos \left( {\theta \left( i \right)} \right) \times ran\left( i \right),\,\,Yr\left( i \right) = \sin \left( {\theta \left( i \right)} \right) \times \,\,ran\left( i \right)$$

21 $$\theta \left( i \right) = \beta \times \pi \times ran1$$

22 $$ran\left( i \right) = \theta \left( i \right) + Q \times ran2$$

23 $$\theta \left( i \right) = \frac{2}{\pi }\arccos \frac{1}{3}\arctan \left( {it} \right)$$

24 $$ran\left( i \right) = \frac{1}{2}\arctan \left( {it} \right)$$

In Eq. (22), “$\beta$ refers to constant constraint among [0.5, 2], $Q$ refers to constant constraint among 0.5 to 2, and $ran1$ and $ran2$ refers to 2 arbitrary constraints”.

Proposed Swooping stage: Conservatively, this stage is shown in Eq. (25), however, as per SSU-BES, this stage is computed from the combination of SSO update as in Eq. (27), in which,$sd_{w}$ refers to weighted standard deviation, $\Re 2$ refers to random parameter,$\alpha_{k}$ →inertia coefficient, $k = 1,2,....\,k_{\max } ,\,\left. {\frac{{\partial \left( {obj} \right)}}{\partial \chi j}} \right|_{{\chi_{i,j}^{k} }}$ denote derivative $obj$ at location $\chi_{i,j}^{k}$, velocity →$V$. Further, $sd_{w}$ is computed as in Eq. (27), where, $\mathop{z}\limits^{\rightharpoonup}$→ weight of average bald eagle swing from best position, $z$→ observation value of shark towards prey, $\varpi$→ weight of each observation of prey and $K^{\prime}$→ count of non-zero observation towards prey.

25 $$\begin{gathered} Z_{new} = rand \times Z_{best} + p1\left( i \right) \times \left( {Z_{i} - it1 \times Z_{mean} } \right) \hfill \\ + Y1\left( i \right) \times \left( {Z_{i} - it2 \times Z_{best} } \right) \hfill \\ \end{gathered}$$

26 $$Z_{new} = \left( {\frac{\begin{gathered} rand \times Z_{best} + p1\left( i \right) \times \left( {Z_{i} - it1 \times Z_{mean} } \right) \hfill \\ + Y1\left( i \right) \times \left( {Z_{i} - it2 \times Z_{best} } \right)\, \hfill \\ \end{gathered} }{{\,\left. {\eta_{k} .\Re 1.\frac{{\partial \left( {obj} \right)}}{\partial \chi j}} \right|_{{\chi_{i,j}^{k} }} + \alpha_{k} .\Re 2.V_{i,j}^{k - 1} }}} \right)sd_{w}$$

27 $$sd_{w} = \frac{{\sum\nolimits_{i = 1}^{K} {\varpi_{i} \left( {z_{i} - \mathop{z}\limits^{\rightharpoonup} _{\varpi } } \right)} }}{{\frac{{\left( {K^{\prime} - 1} \right)\sum\nolimits_{i = 1}^{k} {\varpi_{i} } }}{K}}}$$

28 $$p1\left( i \right) = \frac{pr\left( i \right)}{{\max \left( {\left| {pr} \right|} \right.}},\,\,Y1\left( i \right) = \frac{Yr\left( i \right)}{{\max \left( {\left| {Yr} \right|} \right.}}$$

29 $$pr\left( i \right) = ran\left( i \right) \times \sinh \left( {\theta \left( i \right)} \right),\,\,Yr\left( i \right) = ran\left( i \right) \times \cosh \left( {\theta \left( i \right)} \right)\,\,$$

30 $$\theta \left( i \right) = \beta \times \pi \times ran3,\,\,\,ran\left( i \right) = \theta \left( i \right)$$

## Results and discussion

### Simulation set up

This work was executed in MATLAB. The betterment of Tri classifier + SSU-BES was computed with data in [40]. The HC+SMA-SSA was evaluated with SVM [2], TQWT [9], Tri classifier + SSO, Tri classifier + BES, Tri classifier + DHOA, Tri classifier + BWO, Tri classifier + HHO and LSTM, DBN, CNN and RNN. The analysis was done for two cases such as valence and arousal.

### Dataset description

We took a multimodal dataset for the analysis of human affective states. The electroencephalogram (EEG) and peripheral physiological signals of 32 participants were recorded as each watched 40 one-minute long excerpts of music videos. Participants rated each video in terms of the levels of arousal, valence, like/dislike, dominance and familiarity. The data was recorded in two separate locations. Participants 1-22 were recorded in Twente and participant 23-32 in Geneva. Due to a different revision of the hardware, there are some minor differences in the format. First, the order of EEG channels is different for the two locations. Second, the GSR measure is in a different format for each location. The DEAP dataset can be accessed from: https://www.eecs.qmul.ac.uk/mmv/datasets/deap/download.html.

All metadata contained in four spreadsheets (online ratings, video list, participant ratings and participant questionnaire). The data are collected from 32 participants. Each participant file contains two arrays like data and labels. The size of the data array is 40x40x8064 (i.e. video/trial x channel x data) where the size of label is 40x4 (i.e. video/trial x label). Four labels are considered in the dataset such as valence, arousal, dominance and liking. Moreover, the data was down sampled to 128Hz. And, it was segmented into 60 second trails and 3 second pre-trail baseline removed. The trial was reordered from presentation order to video (i.e. experiment id) order. Herein, two labels such as arousal and valence are considered.

### Performance

**Fig. 3:**
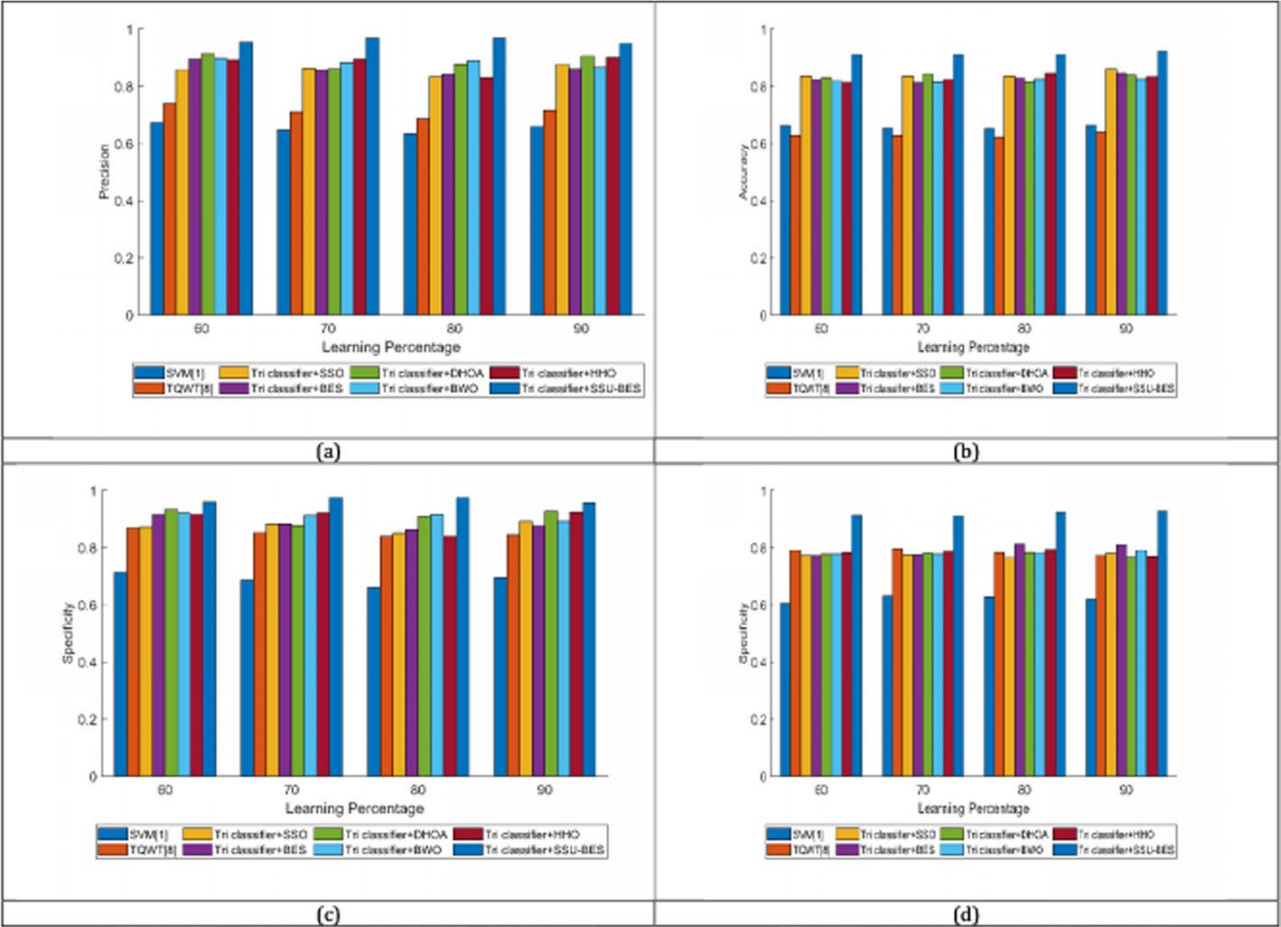
Investigation on Tri classifier + SSU-BES over existing schemes for **a** Precision, **b** accuracy, **c** specificity and **d** sensitivity for valence case

**Fig. 4:**
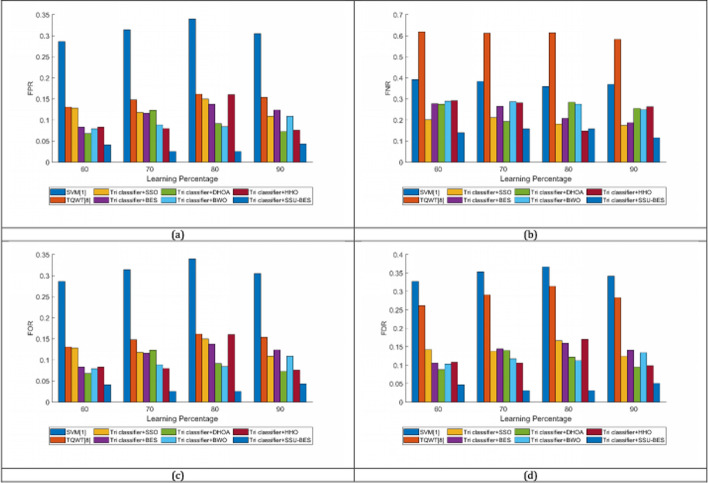
Investigation on Tri classifier + SSU-BES over existing schemes for **a** MCC, **b** NPV, **c** F1-score for valence case

**Fig. 5:**
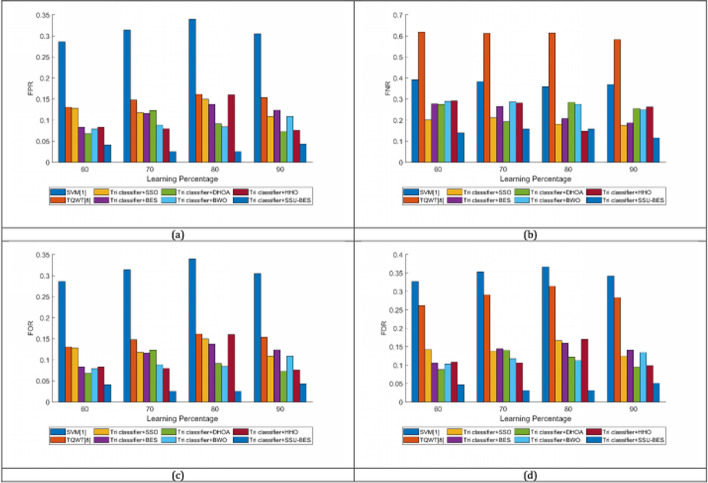
Investigation on Tri classifier + SSU-BES over existing schemes for **a** FPR, **b** FNR, **c** FOR and **d** FDR for valence case

**Fig. 6:**
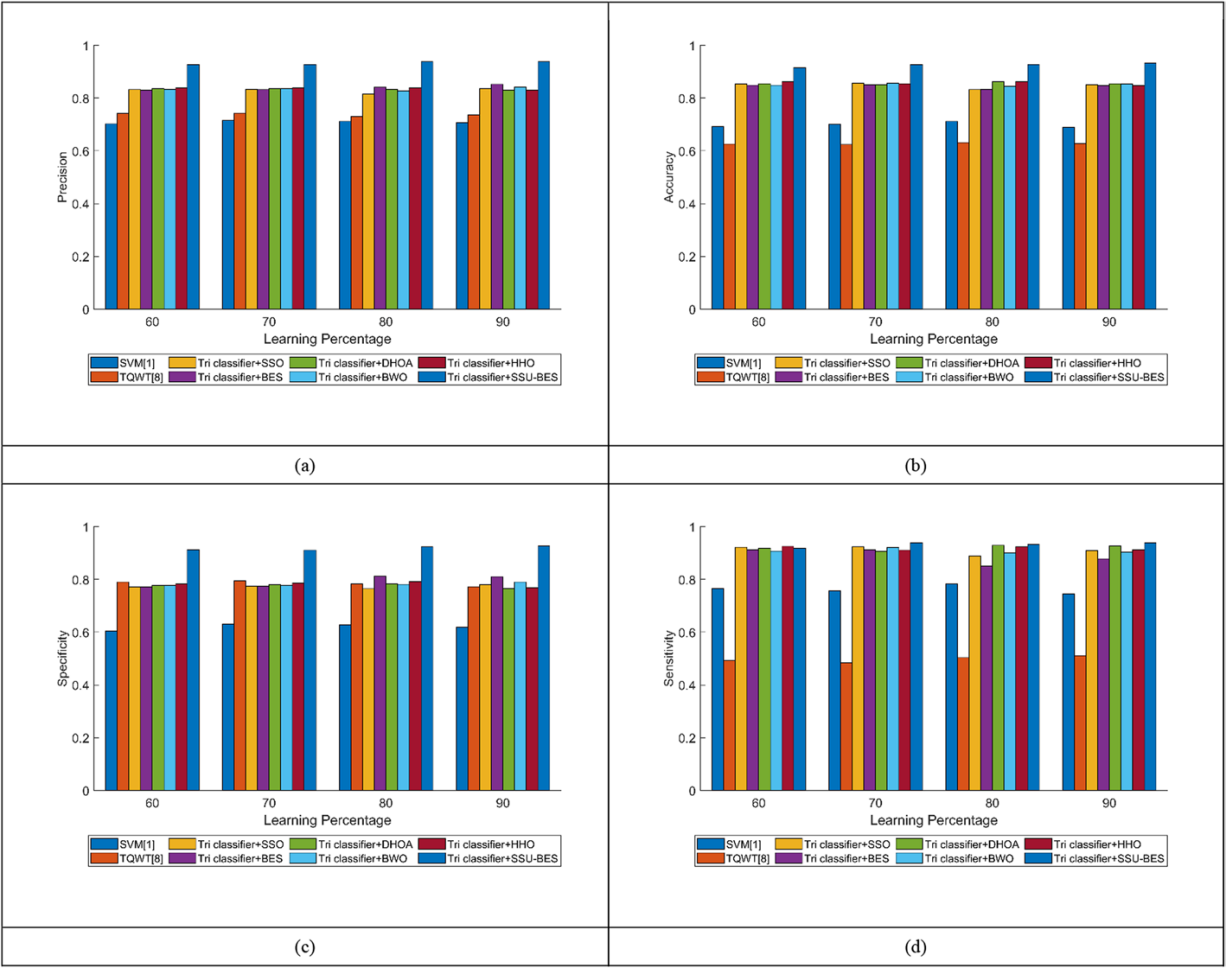
Investigation on Tri classifier + SSU-BES over existing schemes for **a** Precision, **b** accuracy, **c** specificity and **d** sensitivity for arousal case

**Fig. 7:**
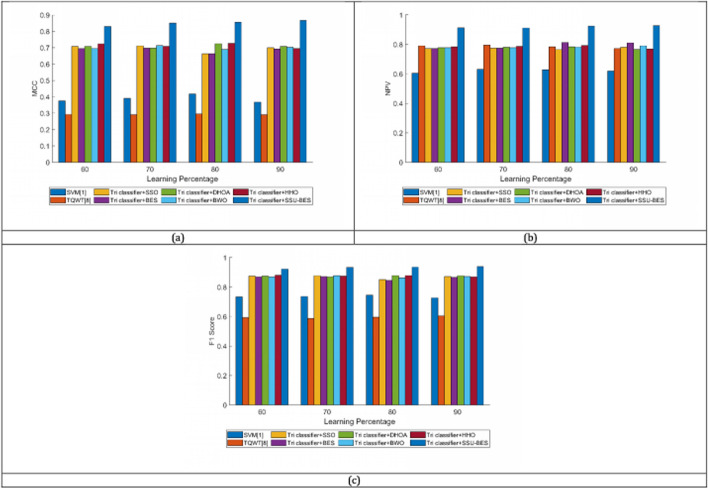
Investigation on Tri classifier + SSU-BES over existing schemes for **a** MCC, **b** NPV, **c** F1-score for arousal case

**Fig. 8:**
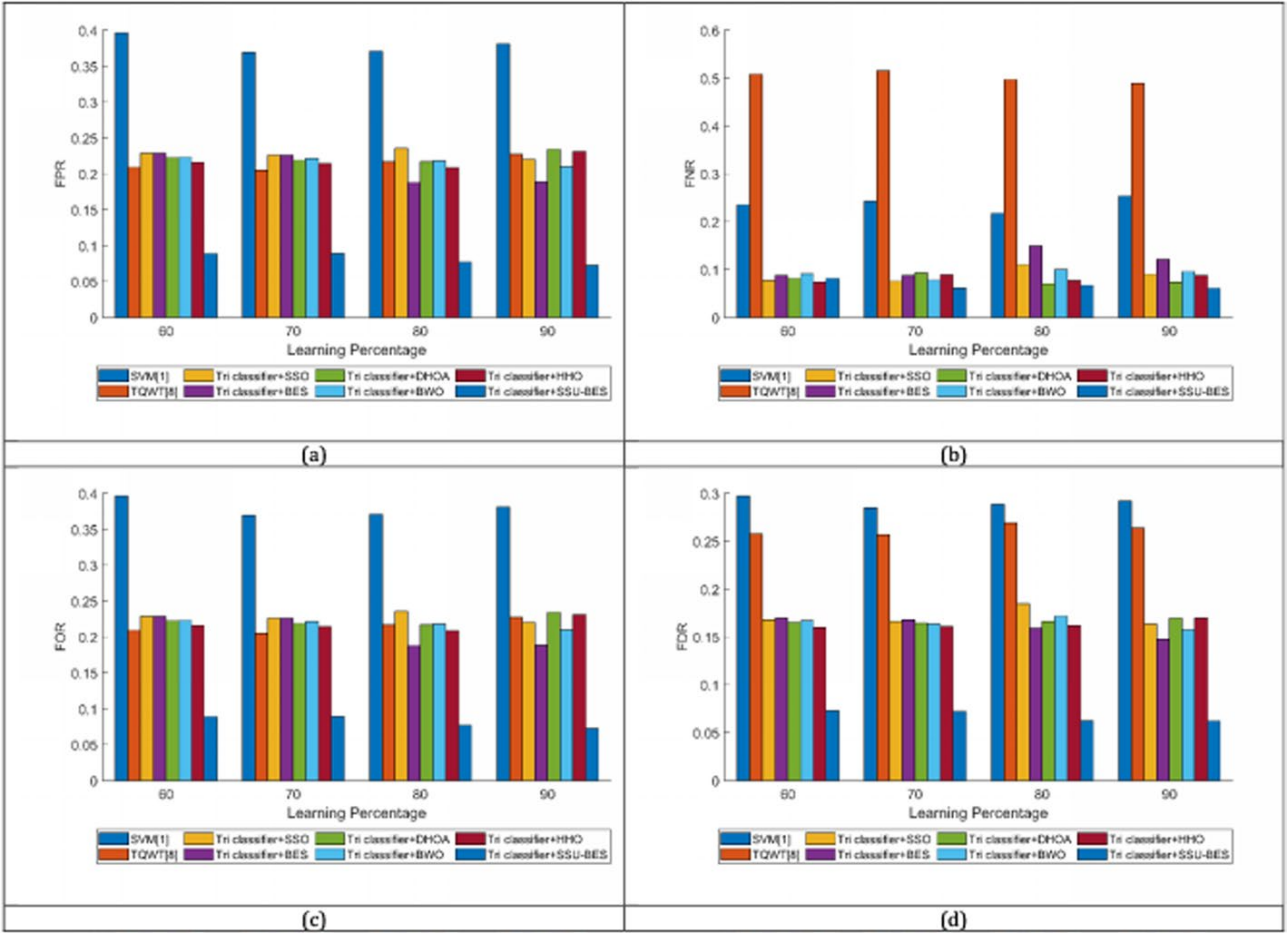
Investigation on Tri classifier + SSU-BES over existing schemes for **a** FPR, **b** FNR, **c** FOR and **d** FDR for arousal case

The study on Tri classifier + SSU-BES model is computed over SVM [19], TQWT [26], Tri classifier + SSO, Tri classifier + BES, Tri classifier + DHOA, Tri classifier + BWO, Tri classifier + HHO and LSTM, DBN, CNN and RNN. The estimation of Tri classifier + SSU-BES based EEG emotion detection done over SVM [19], TQWT [26], Tri classifier + SSO, Tri classifier + BES, Tri classifier + DHOA, Tri classifier + BWO, Tri classifier + HHO are exposed in Figs. 3, 4, 5 for valence case and Figs. 6, 7, 8 for arousal case. The analysis of Tri classifier + SSU-BES over varied classifiers such as SVM [2], TQWT [9], LSTM, DBN, CNN and RNN is shown in Tables 1, 2 for valence case and arousal case. Here, Tri classifier + SSU-BES have accomplished high values for positive metrics, while, less values for negative metrics. The FDR attained by Tri classifier + SSU-BES at 80^th^ LR is lesser than FDR attained by Tri classifier + SSU-BES at other LRs for valence case. The FNR attained by Tri classifier + SSU-BES at 90^th^ LR is lesser than FNR attained by Tri classifier + SSU-BES at other LRs for valence case. The MCC for both cases is high at 90^th^ LR. Here as we have done enhancements in DBN and entropy, the resultants of our method looks superior over compared ones. In valence and arousal cases, Tri classifier + SSU-BES is better than compared ones, which can be proven from the classifier analysis in Tables 1 and 2. The improvements in recognition is purely relies with the inclusion of optimal training of classifiers, as the process takes complete responsibility on proper training with the features. Moreover, the improvement in the entropy feature is also another aspect to get the accurate results.

**Table 1:** Analysis on classifiers using valence case

Metrics	Tri classifier + SSU-BES	SVM [2]	TQWT [9]	LSTM	DBN	CNN	RNN
FDR	0.050877	0.34122	0.28333	0.12371	0.14041	0.094488	0.13433
Sensitivity	0.88399	0.63107	0.41748	0.82524	0.8123	0.74434	0.75081
NPV	0.95659	0.69486	0.84592	0.89124	0.87613	0.92749	0.89124
Specificity	0.95659	0.69486	0.84592	0.89124	0.87613	0.92749	0.89124
FPR	0.043413	0.30514	0.15408	0.10876	0.12387	0.072508	0.10876
F1-Score	0.9154	0.64463	0.52761	0.85	0.83527	0.81705	0.80416
FOR	0.043413	0.30514	0.15408	0.10876	0.12387	0.072508	0.10876
Accuracy	0.92188	0.66406	0.63906	0.85938	0.84531	0.83906	0.82344
MCC	0.84484	0.32666	0.29274	0.71902	0.69067	0.68619	0.65031
Precision	0.94912	0.65878	0.71667	0.87629	0.85959	0.90551	0.86567
FNR	0.11601	0.36893	0.58252	0.17476	0.1877	0.25566	0.24919

**Table 2:** Analysis on classifiers using arousal case

Metrics	Tri classifier + SSU-BES	SVM [2]	TQWT [9]	LSTM	DBN	CNN	RNN
Sensitivity	0.93913	0.74576	0.5113	0.84463	0.82392	0.98305	0.83333
FDR	0.062229	0.29223	0.26423	0.17403	0.10469	0.30952	0.31395
Precision	0.93777	0.70777	0.73577	0.82597	0.89531	0.69048	0.68605
FPR	0.072881	0.38112	0.22727	0.22028	0.11417	0.54545	0.47203
F1-Score	0.93845	0.72627	0.60333	0.8352	0.85813	0.81119	0.75255
MCC	0.86636	0.36766	0.29029	0.62624	0.7072	0.53183	0.38257
FNR	0.06087	0.25424	0.4887	0.15537	0.17608	0.016949	0.16667
Specificity	0.92712	0.61888	0.77273	0.77972	0.88583	0.45455	0.52797
NPV	0.92712	0.61888	0.77273	0.77972	0.88583	0.45455	0.52797
Accuracy	0.93359	0.68906	0.62813	0.81563	0.85225	0.74687	0.69688
FOR	0.072881	0.38112	0.22727	0.22028	0.11417	0.54545	0.47203

### Statistical study

Tables 3 and 4 depicts the statistical analysis (cost) by means of novel Tri classifier + SSU-BES oriented model over SVM [19], TQWT [26], Tri classifier + SSO, Tri classifier + BES, Tri classifier + DHOA, Tri classifier + BWO, Tri classifier + HHO. “The meta heuristic schemes are stochastic, and to substantiate its fair evaluation, each model is analyzed quite a lot of times to accomplish high accuracy”. For mean case, a high accuracy of 0.956is gained using with Tri classifier + SSU-BES, while SVM [2], TQWT [9], Tri classifier + SSO, Tri classifier + BES, Tri classifier + DHOA, Tri classifier + BWO, Tri classifier + HHO have acquired less accuracy of 0.69486, 0.84592, 0.89124, 0.87613, 0.92749, 0.89124 and 0.92447 for valence case. Likewise, for arousal case, the Tri classifier + SSU-BES have acquired high accuracy for mean scenario. Thus, it is proved that the proposed optimization strategy gives high efficiency on solving the optimization issue with respect to accurate recognition. The individual improvement in DBN and entropy features adds additional efficiency to get the accurate recognition of emotions.

**Table 3:** Statistical study for valence case

Metrics	SVM [2]	TQWT [9]	Tri classifier + SSO	Tri classifier + BES	Tri classifier + DHOA	Tri classifier + BWO	Tri classifier + HHO	Tri classifier + SSU-BES
Standard Deviation	0.17689	0.25477	0.36446	0.34908	0.36945	0.34208	0.36528	0.43297
Variance	0.031289	0.064906	0.13283	0.12186	0.13649	0.11702	0.13343	0.18746
Mean	0.5123	0.49631	0.5844	0.57921	0.57657	0.57072	0.57479	0.60746
Best	0.69486	0.84592	0.89124	0.87613	0.92749	0.89124	0.92447	0.95659
Worst	0.30514	0.15408	0.10876	0.12387	0.072508	0.10876	0.075529	0.043413

**Table 4:** Statistical study for arousal case

Metrics	SVM [2]	TQWT [9]	Tri classifier + SSO	Tri classifier + BES	Tri classifier + DHOA	Tri classifier + BWO	Tri classifier + HHO	Tri classifier + SSU-BES
Standard Deviation	0.18996	0.21909	0.3315	0.33749	0.3309	0.33514	0.32686	0.43286
Variance	0.036085	0.047998	0.10989	0.1139	0.10949	0.11232	0.10683	0.18737
Mean	0.52573	0.50198	0.58391	0.58238	0.58533	0.58434	0.58291	0.61258
Best	0.74576	0.77273	0.9096	0.87853	0.92655	0.90395	0.91243	0.93913
Worst	0.25424	0.22727	0.090395	0.12147	0.073446	0.096045	0.087571	0.06087

### Convergence study

**Fig. 9:**
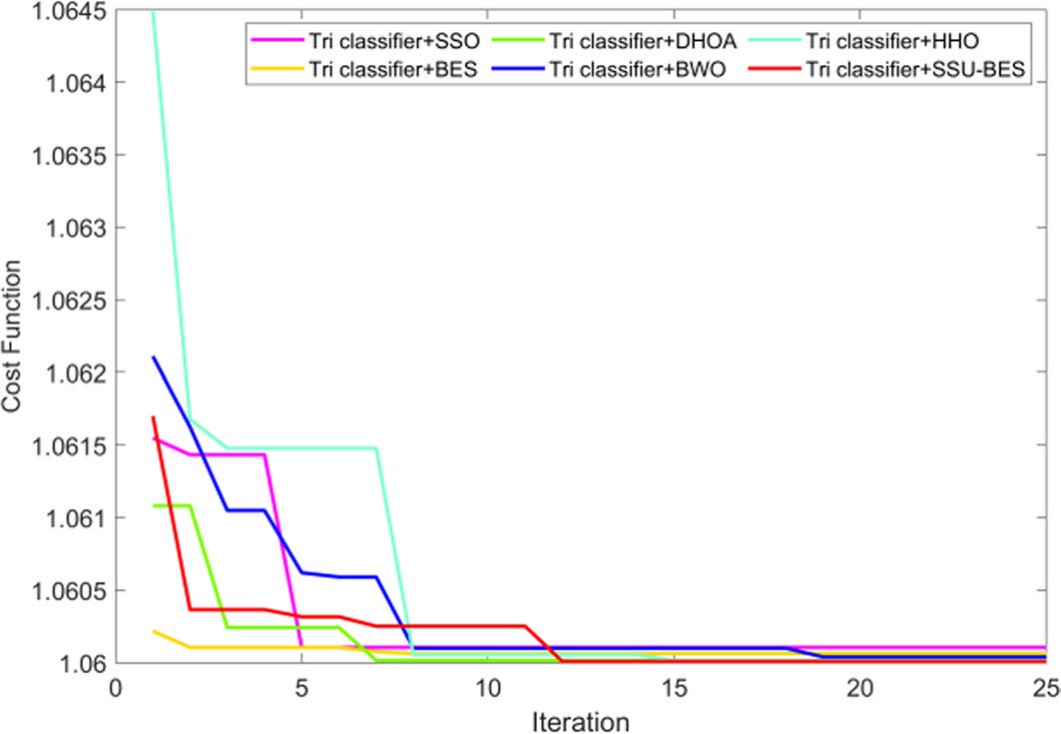
Convergence analysis of SSU-BES technique over compared ones

The cost examination of SSU-BES technique over SSO, BES, DHOA, BWO and HHO is exposed in Fig. 9. This is because, optimization plays a vital role in this work, and the cost was analyzed for varied iterations. In particular, a less cost of 1.06 is acquired by SSU-BES from 12th to 25th iteration. As we have done enhancements in optimization, the approach has resulted in less cost values. Thus, the better convergence is proved by the model. It is clear that, as the iteration increases, the minimal convergence attained. During the initial convergence, even the proposed algorithm shows high error rate. But, as the iterations increased, the error gets minimized, and finally, the least error is obtained by the proposed algorithm.

### Ablation study

The ablation analysis of proposed tri-classifier + SSU-BES is given in Tables 5 and 6. Here, the variation of models with conventional feature and proposed feature is determined. Also, the variation with conventional DBN is also given. A high accuracy of 0.92188 is gained for tri-classifier + SSU-BES that was higher over proposed + conventional entropy, proposed + conventional DBN and proposed + no optimization for valence case. Likewise, FOR metric for proposed work are less than other variants. Finally, it is proved that the proposed concept with improved feature set and improved loss evaluation results with increased accuracy.

**Table 5:** Evaluation of proposed over other features for valence case

Metrics	Tri classifier + SSU-BES	Proposed + Conventional entropy	Proposed + Conventional DBN	No optimization
FDR	0.050877	0.1958	0.41348	0.34395
Sensitivity	0.88399	0.37217	0.84466	0.33333
FOR	0.043413	0.084592	0.55589	0.16314
Accuracy	0.92188	0.65312	0.6375	0.59375
MCC	0.84484	0.34498	0.31351	0.19765
FPR	0.043413	0.084592	0.55589	0.16314
Specificity	0.95659	0.91541	0.44411	0.83686
F1-Score	0.9154	0.50885	0.69231	0.44206
NPV	0.95659	0.91541	0.44411	0.83686
Precision	0.94912	0.8042	0.58652	0.65605
FNR	0.11601	0.62783	0.15534	0.66667

**Table 6:** Evaluation of proposed over other features for arousal case

Metrics	Tri classifier + SSU-BES	Proposed + Conventional entropy	Proposed + Conventional DBN	No optimization
NPV	0.92712	0.91541	0.44411	0.83686
Accuracy	0.93359	0.65312	0.6375	0.59375
FPR	0.072881	0.084592	0.55589	0.16314
Sensitivity	0.93913	0.37217	0.84466	0.33333
MCC	0.86636	0.34498	0.31351	0.19765
FNR	0.06087	0.62783	0.15534	0.66667
Specificity	0.92712	0.91541	0.44411	0.83686
FDR	0.062229	0.1958	0.41348	0.34395
F1-Score	0.93845	0.50885	0.69231	0.44206
Precision	0.93777	0.8042	0.58652	0.65605
FOR	0.072881	0.084592	0.55589	0.16314

## Conclusion

This work proposed an EEG recognition model, where the input signal was pre-processed using Band pass filter. Then, the features like “DWT, band power, spectral flatness, and improved entropy were extracted”. Further, for recognition, tri-classifiers like “(LSTM, improved DBN and RNN)” were used. Also to enhance tri-model classifier performance, the weights of LSTM, improved DBN, and RNN were optimally tuned by the new model named as SSU-BES. The analysis of tri-classifier + SSU-BES over varied classifiers was done for valence case and arousal case. Here, Tri classifier + SSU-BES have accomplished high values for positive metrics, while, less values for negative metrics. The FDR attained by Tri classifier + SSU-BES at 80^th^ LR was lesser than FDR attained by Tri classifier + SSU-BES at other LRs for valence case. The FNR attained by tri-classifier + SSU-BES at 90^th^ LR was lesser than FNR attained by tri-classifier + SSU-BES at other LRs for valence case.

In future, large datasets have to be involved. And, as contrast to the image processing-based method, emotion identification utilizing EEG signals needs a multidisciplinary set of abilities from the fields of engineering, computer science, psychology, and neuroscience. But to move beyond the capabilities of the present algorithms for emotion identification, it is necessary to make new discoveries in neuroscience and psychology or to use a multi-modal strategy that blends EEG-based emotion recognition models with techniques based on picture processing. There is also a need to develop Uncertainty Quantification (UQ) methods that can be used to explain the uncertainty in deep learning models and can handle complex, high-dimensional datasets. EEG does have a very high temporal resolution but a relatively lower spatial resolution. So, the precise classification can be gained by integrating EEG with some higher spatial resolution signals such as NIRS and fMRI.

## Data Availability

The datasets used and/or analyzed during the current study are available from all the authors on reasonable request. The DEAP dataset can be accessed from : https://www.eecs.qmul.ac.uk/mmv/datasets/deap/download.html.

## Abbreviation

ANN: Artificial neural network
BWO: Black widow optimization
BES: Bald eagle search
BiDCNN: Bi-hemisphere discrepancy CNN
BPF: Band pass filter
CFC: Cross-frequency coupling
CNN: Convolutional neural network
DHOA: Deer hunting optimization
DWT: Discrete wavelet transforms
DL: Deep learning
DT: Decision tree
DBN: Deep belief network
EEG: Electroencephalography
FFP: Fractal Firat pattern
GL: Greedy learning
HHO: Harris hacks optimization
k-NN: K-nearest neighbor
HCI: Human–computer interaction
LSTM: Long short term memory
LDA: Linear discriminate analysis
LR: Learning rate
MLF-CapsNet: Multi-level features guided capsule network
ML: Machine learning
MAEL: Mean absolute error loss
RNN: Recurrent neural networks
RFE: Random forest ensemble
RF: Random forest
SSU-BES: Shark smell updated BES optimization
SVM: Support vector machine
SSO: Shark smell optimization
TOPO-FM: Topographic and holographic
TQWT: Tunable Q wavelet transform
WT: Wavelet transform
